# Identification, Characterization and Full-Length Sequence Analysis of a Novel Polerovirus Associated with Wheat Leaf Yellowing Disease

**DOI:** 10.3389/fmicb.2017.01689

**Published:** 2017-09-06

**Authors:** Peipei Zhang, Yan Liu, Wenwen Liu, Mengji Cao, Sebastien Massart, Xifeng Wang

**Affiliations:** ^1^State Key Laboratory for Biology of Plant Diseases and Insect Pests, Institute of Plant Protection, Chinese Academy of Agricultural Sciences Beijing, China; ^2^Laboratory of Phytopathology, University of Liège, Gembloux Agro-Bio Tech Gembloux, Belgium; ^3^National Citrus Engineering Research Center, Citrus Research Institute, Southwest University Chongqing, China

**Keywords:** deep sequencing, wheat, leaf yellowing, *Luteoviridae*, *Polerovirus*, infectious cDNA clone

## Abstract

To identify the pathogens responsible for leaf yellowing symptoms on wheat samples collected from Jinan, China, we tested for the presence of three known barley/wheat yellow dwarf viruses (BYDV-GAV, -PAV, WYDV-GPV) (most likely pathogens) using RT-PCR. A sample that tested negative for the three viruses was selected for small RNA sequencing. Twenty-five million sequences were generated, among which 5% were of viral origin. A novel polerovirus was discovered and temporarily named wheat leaf yellowing-associated virus (WLYaV). The full genome of WLYaV corresponds to 5,772 nucleotides (nt), with six AUG-initiated open reading frames, one non-AUG-initiated open reading frame, and three untranslated regions, showing typical features of the family *Luteoviridae*. Sequence comparison and phylogenetic analyses suggested that WLYaV had the closest relationship with sugarcane yellow leaf virus (ScYLV), but the identities of full genomic nucleotides and deduced amino acid sequence of coat protein (CP) were 64.9 and 86.2%, respectively, below the species demarcation thresholds (90%) in the family *Luteoviridae*. Furthermore, agroinoculation of *Nicotiana benthamiana* leaves with a cDNA clone of WLYaV caused yellowing symptoms on the plant. Our study adds a new polerovirus that is associated with wheat leaf yellowing disease, which would help to identify and control pathogens of wheat.

## Introduction

In 43 countries, wheat (*Triticum aestivum*) is the most important food crop and the primary staple food, feeding at least one third of the world's population. However, wheat yield and quality are seriously impacted by diseases caused by fungi, bacteria, and viruses (Mehta, 2014). Nearly 50 viruses have been reported to infect wheat in the world, resulting in typical symptoms including mosaic, streak, yellowing, dwarfing, and rosette stunting (Lister and Ranieri, 1995; Wang et al., 2010; Rotenberg et al., 2016). Wheat yellow dwarf disease is a recurrent and costly problem throughout China, where it has caused serious epidemics seven times since the 1970 s. In early-planted winter wheat fields, when the densities of populations of aphids are high, yield losses on susceptible cultivars can reach to average 10~15% or even more than 50% in northwestern regions (Wang et al., 2010).

The causal agents of yellow dwarf disease are barley yellow dwarf viruses (BYDVs) and were previously classified into five strains based on epitope profile and aphid vector specificity (Rochow, 1969; Rochow and Muller, 1971). Now that the complete genomes of some of the BYDVs and their genome structures have been reported, the viruses are considered to be different species classified in the family *Luteoviridae*, that infect plants in the family *Poaceae* and are transmitted by aphids (Miller et al., 2002; Krueger et al., 2013). *Luteoviridae* comprise three genera: *Enamovirus, Polerovirus*, and *Luteovirus*, and seven species considered as “unassigned” members according to their genome characterization, nucleotide sequence identity and most efficient vector (Domier, 2012). Viruses in the *Luteoviridae* have linear, positive-sense, 5.5~6-kb RNA genomes with six recognized AUG-initiated open reading frames (ORFs), i.e., ORF 0–5 in poleroviruses and ORF 1–6 in luteoviruses and three or four untranslated regions (UTRs) (Miller et al., 1995). In addition, a small non-AUG-initiated ORF (ORF3a), required for long-distance movement, was predicted in poleroviruses, and luteoviruses through statistical analysis, and confirmed by functional analysis (Smirnova et al., 2015). The cereal-infecting members of *Luteoviridae* comprise BYDV-PAV, -MAV, -PAS, -kerII, and -kerIII species within the genus *Luteovirus*; cereal yellow dwarf virus-RPV (CYDV-RPV, formerly BYDV-RPV), CYDV-RPS and maize yellow dwarf virus-RMV (MYDV-RMV, formerly BYDV-RMV) in the genus *Polerovirus* as well as BYDV-SGV, and wheat yellow dwarf virus-GPV (WYDV-GPV, formerly BYDV-GPV) that have not yet been assigned to any genus (Miller and Rasochová, 1997; Hawkes and Jones, 2005; Luciozavaleta et al., 2007; Zhang et al., 2009; Domier, 2012; Krueger et al., 2013). In China, four species of *Luteoviridae* infect cereals according to Rochow's system of classification: BYDV-GAV, -PAV, MYDV-RMV, and WYDV-GPV (Zhou et al., 1987; Liu, F. et al., 2007; Wu et al., 2011). BYDV-GAV is very similar to BYDV-MAV (Jin et al., 2004; Zhang et al., 2009), WYDV-GPV is closely related to CYDV-RPV (Zhang et al., 2009), while BYDV-PAV-CN is highly diverged from the other BYDVs (Liu, F. et al., 2007; Wu et al., 2011).

Traditionally, viruses have been detected and identified using biological, electron microscopy, serological, and molecular biological methods, which depend on the development of antibodies or knowledge of sequences of potential pathogens. Next-generation sequencing (NGS, or deep sequencing), however, provides a powerful alternative for virus detection. The advantage of this method is that it no need prior knowledge of the host or viral information and can detect both RNA and DNA virus. When plants infected by any kinds of viruses, the small interfering RNAs (siRNAs) are generated and accumulated to respond to antiviral defense (Hamilton and Baulcombe, 1999; Mlotshwa et al., 2008). This defense is initiated by cleavage of viral dsRNA into viral derived siRNA (vsiRNA) by a Dicer family member (Voinnet, 2001; Baulcombe, 2004). Having noted that vsiRNA are often overlapping, it is possible to identify a virus through sRNA sequencing followed by assembly of sRNAs into a partial or even complete viral genome (Mierlo et al., 2010).

Since the early 1980s in China, a nationwide survey for wheat yellow dwarf disease has targeted multiple commercial field sites in the main wheat-growing regions where epidemics have been reported. RT-PCR, enzyme-linked immunosorbent assays (ELISA), and dot-blot hybridization were used to determine the occurrence of different luteoviruses in China (Liu, Y., et al., 2007). In the past, BYDV-GAV, -PAV or WYDV-GPV were detected in more than 80% of wheat samples that had yellowing and dwarfing symptoms, but this percentage dropped to 50% in more recent years (Zhao et al., 2010). Because we suspected that other novel viruses might be associated with wheat samples showing dull yellowing, dwarfing, and excessive tillering symptoms, next-generation sequencing was used to detect any viruses in a wheat sample collected from Jinan, in Shandong Province of China. As a result, we identified a new virus, temporarily named wheat leaf yellowing-associated virus (WLYaV).

## Materials and methods

### Plant material

During field surveys in April 2016, 12 wheat samples exhibiting flag leaf yellowing and little dwarfing were collected from Jinan, Shandong Province of China. Whole plants were put into plastic bags and their roots kept moist during transport to the laboratory. One hundred milligrams of fresh leaves were cut from the plants to extract total RNA; other leaves were stored at −70°C. Healthy wheat plants growing in an insect-proofed greenhouse served as controls.

### RNA extraction and detection of known viruses

Total RNA of wheat leaves was extracted using TRIzol reagent according to the manufacturer's instruction (Invitrogen, USA). The quality of RNA was tested using a Nanodrop 2,000 spectrophotometer (Thermo Fisher Scientific, Waltham, MA, USA), and RNA integrity was verified by gel electrophoresis.

BYDV-GAV, -PAV and WYDV-GPV were detected by RT-PCR using specific primers detailed in Table S1, which also gives the sequences of all primers used in this study. B/W-YDV-infected plants that were used as positive controls were growing in the growth chamber (16 h light at 20°C/8 h dark at 18°C). Reverse transcription was performed in a total volume of 20 μL with 1 μg of RNA, 2 μL of reverse (R) primer (10 μM), 2 μL of dNTP Mix (each 2.5 mM; TaKaRa, China), 4 μL of 5× M-MLV buffer (Promega, USA), 1 μL of M-MLV reverse transcriptase (200 U/μL; Promega), 0.5 μL of Recombinant RNase Inhibitor (40 U/μL; TaKaRa) and DEPC water. The PCR contained 2 μL of cDNA, 2.5 μL of 10× buffer (Mg^2+^, 15 mM, TaKaRa), 2 μL of dNTP Mix (each 2.5 mM; TaKaRa), 0.5 μL of forward (F) and reverse (R) primer (10 μM), 0.2 μL of rTaq (5 U/μL; TaKaRa) and 17.3 μL of ddH_2_O. The PCR reaction was performed using thermal cycler (Bio RAD, Hercules, CA, USA) as follows: denaturation at 94°C for 3 min; 35 cycles at 94°C for 30 s, 58°C for 45 s and 72°C for 80 s; final extension at 72°C for 10 min (Zhao et al., 2010). PCR products were electrophoresed in 1% agarose gel and stained with ethidium bromide (EB).

### Small RNA (sRNA) sequencing

The quality of extracted RNA was tested using the Nanodrop 2000 spectrophotometer (Thermo Fisher Scientific) and Bio Analyzer 2100 (Agilent Technology, Santa Clara, CA, USA). An RNA sample with an RIN (RNA integrity number) value ≥6 was considered as high enough quality and used in the next steps. The small RNAs (sRNAs) were separated using PEG8000 and ligated to a 3′ adaptor. The 36~44-nt (ligated sRNAs) bands were purified using PAGE in a 15% denatured gel and linked to 5′ adaptor. The first-strand cDNA was synthesized using the pair-linked sRNAs, and 16 cycles of PCR amplification were performed. The 140~160-bp products, namely the sRNAs library, were purified by 3.5% agarose gel electrophoresis. The library was quantified by ECO (Illumina, San Diego, CA, USA) and submitted for sequencing on the Illumina 2,500 platform (Illumina).

### Analysis and assembly of sRNA data

Raw reads from the Illumina platform were processed to trim adaptor sequences and low-quality reads. Unique sequences were then generated as clean reads by collapsing the identical sequences. The abundance of sRNAs was determined using the software Bowtie2 and default parameters (Langmead and Salzberg, 2012). Then the clean reads were assembled using the Velvet program with a *k*-mer of 17 as the minimal overlapping length required for joining sRNAs into larger contigs (Zerbino and Birney, 2008). Assembled contigs were screened against the GenBank nucleotide collection (nt) and non-redundant protein sequences (nr) databases using a BlastN and BlastX search using standard parameters respectively (https://blast.ncbi.nlm.nih.gov/Blast.cgi).

### Validation and completion of viral genome

Contigs and gaps were confirmed and filled by RT-PCR using specific primer pairs 1, 2, 3, 4, 1-2, 2-3, and 3-4 F/R (Table S1) designed according to the consensus sequences of the assembled viral contigs with more than 70-nt overlapping. The sugarcane yellow leaf virus (ScYLV, AF157029.1) genome sequence was used for the alignment and positioning of the contigs. The terminal sequences of the viral genome were obtained using 5′ and 3′ rapid amplification of cDNA ends (RACE) kits according to the manufacture (Invitrogen). The PCR products were collected using Wizard SV Gel and PCR Clean-Up System (Promega). The purified PCR products were then cloned into the pEASY-T5 vector (TransGen Biotech, China) and used to transformed Trans-T1 competent cells (TransGen Biotech) following the manufacturers' instructions. The clones harboring the transformed vector were identified by PCR and Sanger sequencing (Sangon Biotech (Shanghai) Co., China). The results of sequencing were assembled using DNAMAN (version 6) program (Lynnon Biosoft, San Ramon, CA, USA) with more than 70-nt overlapping regions to form the full-length viral genome.

### Analysis of the viral genome and sRNA

After obtaining the whole viral genome, open reading frames (ORFs) were predicted using SnapGene software (Ian, 2004). The distribution and coverage of vsiRNA was determined using the software Bowtie2 under default parameters, and the results were exported to Excel (Microsoft, Redmond, WA, USA) for further analysis. Contigs were mapped to the WLYaV genome using the CLC Genomics Workbench (Qiagen, Valencia, CA, USA).

### Identity calculations and phylogenetic analyses

The identities of nucleotide sequences of the whole genome, untranslated regions and deduced amino acid sequences of seven ORFs of WLYaV with other viruses belonging to the family *Luteoviridae* (Table S2) that were retrieved from NCBI (http://www.ncbi.nlm.nih.gov/) were calculated by the Needle program (Liu et al., 2009). Sequences were aligned with the ClustalW method, and phylogenetic trees were constructed by the neighbor joining method using MEGA 6 software (Tamura et al., 2013). The reliability of each branch was evaluated with bootstrap (1000 repeats).

### Agrobacterium mediated infectious cDNA clone

The whole nucleotide sequence of WLYaV was divided into two overlapping parts, A (nt 1–3 059) and B (nt 3 035–5 772), a *Nco*I restriction enzyme site was within the overlapping region (nt 3 045–3 050). A and B were amplified by RT-PCR with specific primer pairs (A-Stu-F/A-Nco-R, B-Nco-F/B-Sal-R, respectively), adding restriction enzyme sites *Stu*I and *Sal*I, respectively. The amplification products of fragments A and B were purified and cloned into the pEASY-T5 vector and sequenced as above. pCB301 vector (kindly supplied by Prof. Xiaorong Tao, Nanjing Agricultural University), a binary vector with 2 × 35S promoter, ribozyme and NOS terminator (Shen et al., 2014; Wang et al., 2015), and plasmids containing fragment A and B without mutations were digested with restriction enzymes and ligated using T4 ligase (Promega, USA) to produce pCB301-WLYaV. Plasmid pCB301 (as a negative control) and pCB301-WLYaV were transferred to separate suspensions of *Agrobacterium tumefaciens* strain GV3101 (Wang et al., 2015). Then the abaxial surface of 2-week-old *Nicotiana benthamiana* leaves (3~4 leaves) was infiltrated with the bacterial cultures (Chen et al., 2016). At 14 days post inoculation (dpi), systemic virus dissemination was evaluated in non-inoculated leaves by RT-PCR using specific primer pair CP-F/R (Table S1).

## Results

### Detection of known luteoviruses in wheat samples

**Figure 1:**
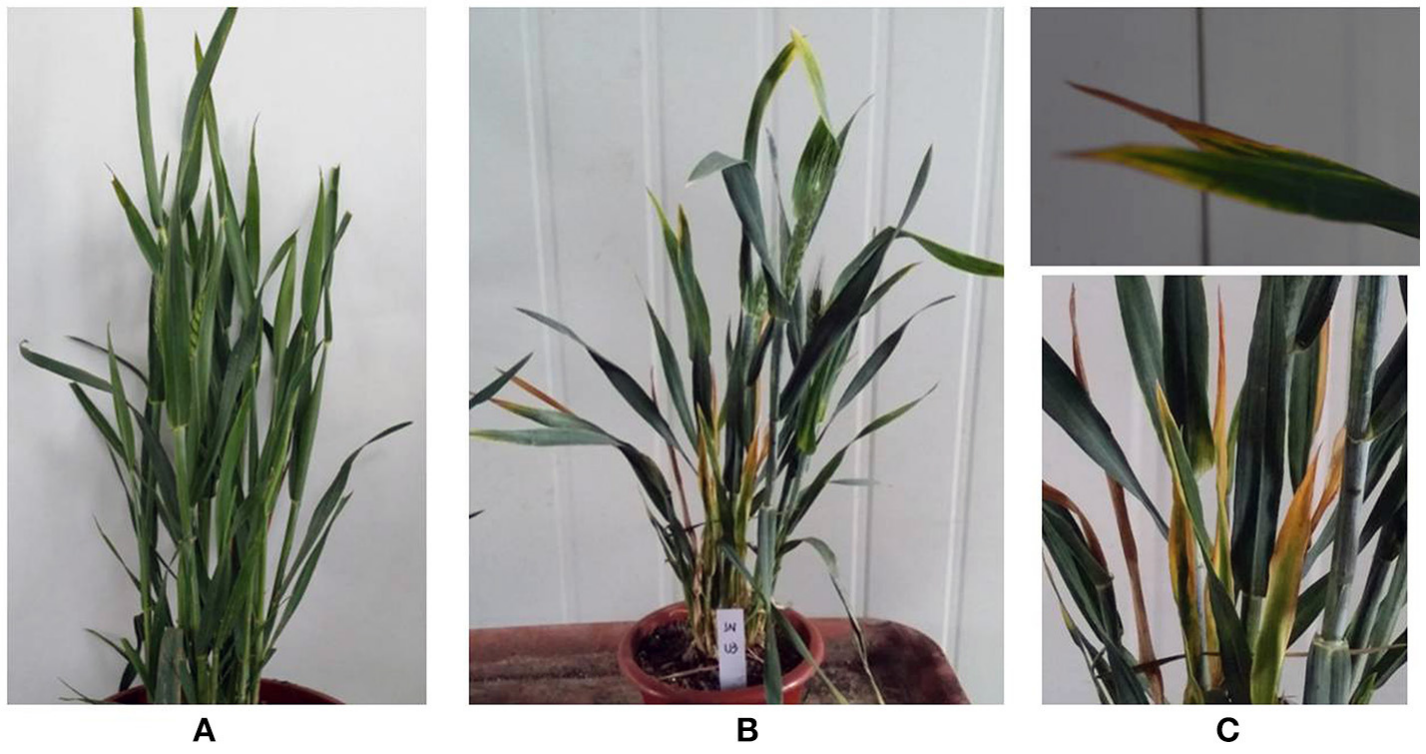
Symptoms on wheat samples collected from Jinan, China. **(A)** Symptomless, apparently healthy sample. **(B,C)** Sample JN-U3 infected with virus newly discovered, with dull yellowing and little dwarfing.

In 12 wheat samples from Jinan, China that had yellow dwarf symptoms, three known B/W-YDVs (-GAV, -PAV and -GPV) in China were detected. Eight samples were infected with one of the BYDVs, and none had a mixed infection (Table S3). None of the above three viruses were detected in sample JN-U3, which exhibited dull yellowing on the leaf margins and little dwarfing (Figures 1B,C) comparing with the healthy sample (Figure 1A), while bright yellowing was found on samples infected by B/W-YDVs. Therefore, sample JN-U3 was used for the next experiment.

### Analysis and assemble of sRNA library

**Figure 2:**
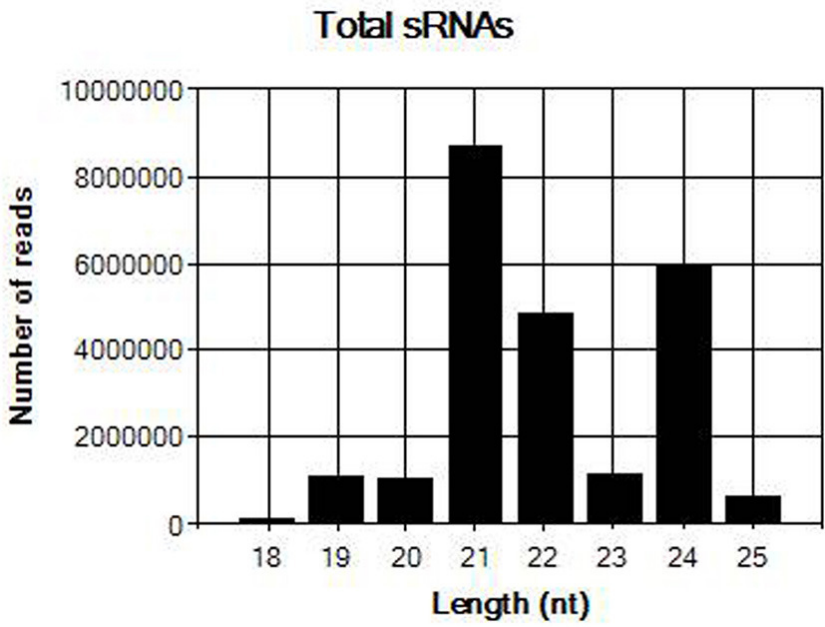
Length distribution of total sRNAs in sample JN-U3.

The sRNA library from sample JN-U3 was constructed and sequenced using Illumina Hiseq 2,500 platform. In total, 25,605,387 clean reads that had passed quality control were produced after removing adaptor sequences and low-quality reads. The length of the clean reads ranged from 18 to 25 nt (Figure 2); 21-nt reads were the most abundant, followed by 24 and 22-nt reads. After assembly using the Velvet program, 8,798 contigs were generated.

### Identification and sequencing of unknown viral genome

After blast analysis of the assembled contigs, four contigs of 1,151, 701, 718, and 1,714 nucleotides, respectively, had high identities with several viruses in the family *Luteoviridae*, and may be associated with the yellowing symptom. The higher identities were observed with ScYLV, with 83% identity on 99% coverage of the sequence for contig 3, for example. Four contigs were aligned to different positions of the ScYLV genome, and their relative positions and gaps were predicted. RT-PCR and RACE-PCR were performed to confirm the contig sequences, fill gaps and obtain the terminal sequences (Figure S1). The RACE-PCR was repeated three times with the similar results. Finally, the assembled genome of this virus was constituted by 5,772 nt, and deposited in GenBank as accession KY605226. The other 11 samples from Jinan were negative for WLYaV (Table S3) using RT-PCR and primer CP-F/R (Table S1).

### Characterization of the viral genome and sRNAs

**Figure 3:**
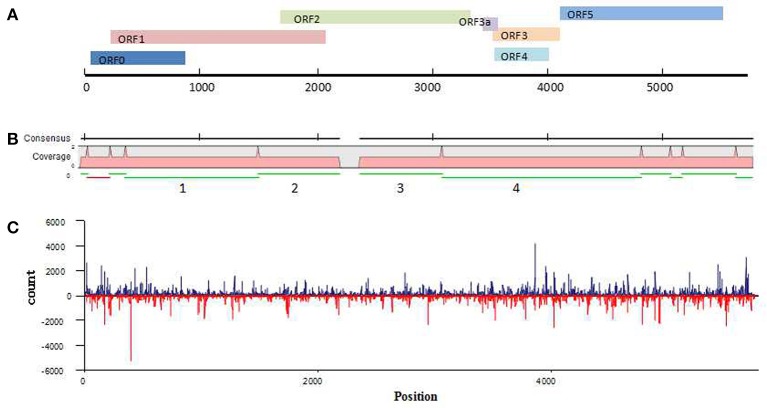
Analysis of WLYaV genome. **(A)** Characteristics of WLYaV genome, full length: 5,772 nt; 5′UTR: nt 1~58, with ACUAAA start; ORF0: nt 59~877, encodes a 31.4 kD protein (P0), a putative RNA-silencing suppressor (RSS); ORF1: nt 228~2,099, encodes a 69.1 kD putative polyprotein (P1); ORF2: nt 1,658~3,364, encodes a putative fusion protein of RdRp (P1–P2 fusion) by a −1 ribosomal frameshift with ORF1; ORF3a: nt 3,435~3,569, encodes a protein that may be involved in long-distance movement; ORF3: nt 3,553~4,143, encodes a 21.7 kD protein, a putative coat protein (P3); ORF4: nt 3,584~4,036, within ORF3, encodes a 17.0 kD protein, a putative movement protein (P4); ORF5: nt 4,144~5,577, encodes a putative fusion protein, a read-through protein (P3–P5 fusion) with ORF3 by suppression of termination; 3′UTR: nt 5,578~5,772, without a polyA tail. **(B)** Contigs mapped to the WLYaV genome: 11 of 8,798 contigs were remapped to the WLYaV genome, which covered 99% of the genome; the four numbered contigs were positioned using a blast search of known viruses. **(C)** Hotspots along the WLYaV genome, blue: positive strand, red: reverse strand. 5.25% of total sRNAs reads were derived from WLYaV, which were well-scattered on the viral positive and reverse strands except for several peaks at ORF0 (RSS), ORF3 (CP), and ORF5 (RTD).

Because the newly discovered virus was found in a wheat sample that had dull yellowing and little dwarfing symptoms, the virus has been temporarily named wheat leaf yellowing-associated virus (WLYaV). Six ORFs (ORF0, ORF1, ORF2, ORF3, ORF4, ORF5) were predicted by SnapGene software, and 5′ and 3′ UTRs, one intergenic-UTR (I-UTR) and ORF3a were also found in the viral genome (Figure 3A), suggesting the genome organization is similar to that of viruses in the genus *Polerovirus* (Miller et al., 1995). The 5′ UTR consists of 58 nt starting with ACUAAA, while the conserved sequence that start the 5′ end of poleroviruses is ACAAAA (Guilley et al., 1994; Moonan et al., 2000; Mo et al., 2010). ORF0 (nt 59~877) is the first ORF encoding a 31.4 kD protein (P0), which is a putative RNA-silencing suppressor (RSS) (Pfeffer et al., 2002, 2017; Kozlowska-Makulska et al., 2010). A putative F-box like motif (IPIIL) conserved among poleroviruses (LPxxL/I) has been found (Figure S2), which is required for RSS function (Pazhouhandeh et al., 2006). ORF1 (nt 228~2,099) encodes a 69.1 kD putative polyprotein (P1) with a proteinase motif that can generate three proteins including the viral genome-linked protein (VPg) (Figure S2) (Miller et al., 2002; Nickel et al., 2008). ORF2 (nt 1,658~3,364) and ORF1 compose a putative fusion protein of RdRp (P1-P2 fusion) by a -1 ribosomal frameshift at nt 1,658, which is regulated by a GGGAAAC sequence at nt 1,658~1,664 within the overlapping region of ORF1 and ORF2 and is involved in the conserved GDD amino acid core (Figure S2) (Prüfer et al., 1992; Nixon et al., 2002). ORF2 is followed by a 188 nt I-UTR (nt 3,365~3,552). This sequence contains the ORF3a starting with a putative CUG initiation codon (nt 3,435~3,569; 4.91 kD), which encodes a protein that involved in the viral long-distance movement (Smirnova et al., 2015). ORF3a, ORF3~ORF5 might be expressed through the 3′ co-terminal sgRNAs, yielding the putative capsid, CP-RTD (RTP) and movement proteins (Hwang et al., 2013). ORF3 (nt 3,553~4,143) encodes a 21.7 kD protein of putative coat protein (P3). ORF4 (nt 3,584~4,036) present within ORF3 and encodes a 17.0 kD putative movement protein (P4). ORF5 (nt 4,144~5,577) and ORF3 encode a fusion protein (P3–P5 fusion), a putative readthrough protein that may be involved in virus transmission (Brault et al., 1995). The 3′ UTR consists of 195 nt (nt 5,578~5,772) without a polyA tail.

After the complete viral genome was obtained, total contigs were remapped to the viral genome (Figure 3B). This time, we obtained 11 mapped contigs, which nearly covered the entire genome, while initially only 4 of the 11 contigs had homologies with *Luteoviridae* family. This difference may be due to the low identities of the seven unrecognized contigs with other known viruses.

### Analysis of vsiRNAs

To identify the hotspots of viral siRNAs (vsiRNAs), we analyzed the genome region targeted by RNAi by mapping the sRNAs on the viral genome (Figure 3C). A total of 1 301,797 reads (5.25% of total sRNAs reads) were mapped. They were well-scattered on the viral genome and anti-genome strands except for several peaks at ORF0 (RSS), ORF3 (CP) and ORF5 (RTD). The higher number of siRNAs corresponding to CP and RTD would correspond to a higher representativity of the RNA targets that correspond to both genomic and subgenomic RNA species.

**Figure 4:**
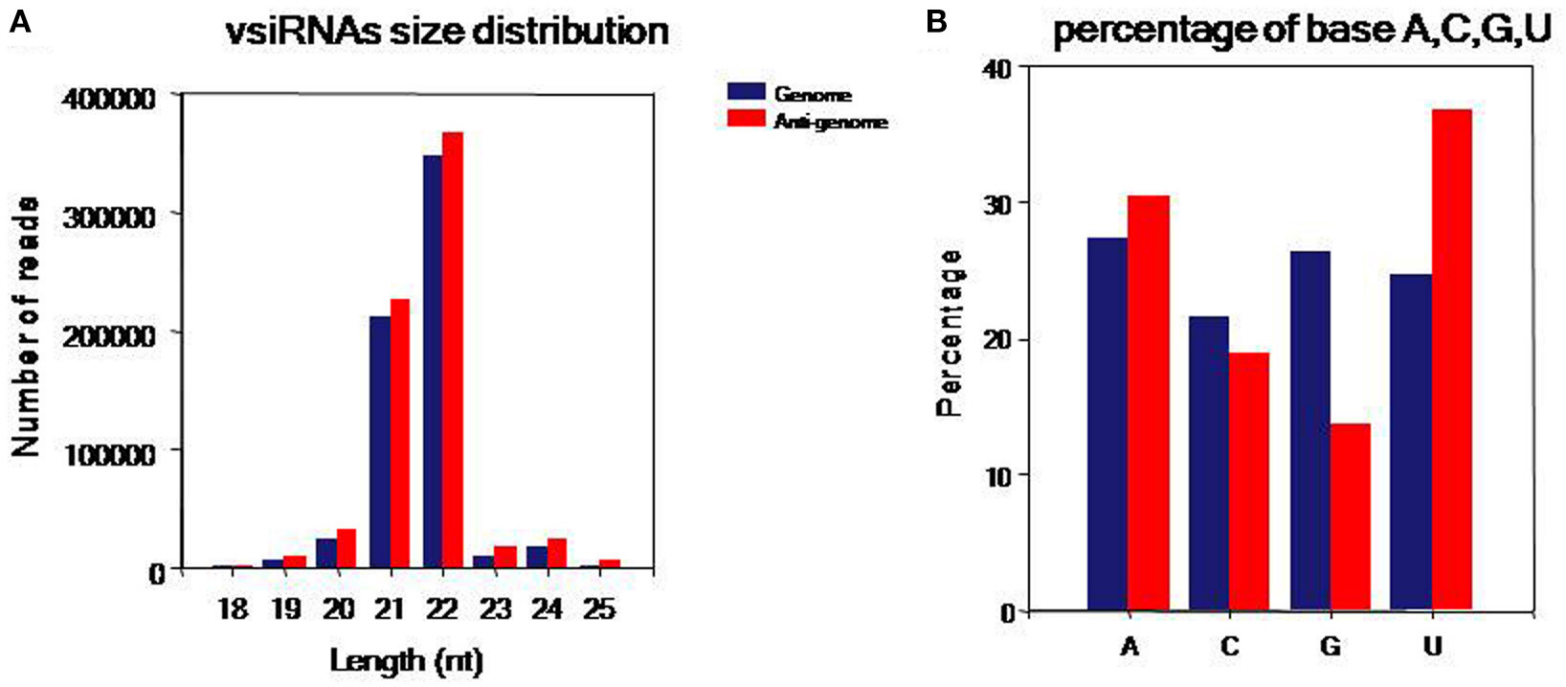
Characteristics of the vsiRNAs. **(A)** Size distribution of vsiRNAs, 18~25 nt long, with 22- and 21-nt sRNAs predominated. **(B)** Percentage of A/U/G/C bases in vsiRNAs, a bias toward bases A and U especially in the anti-genome was found (52.06% in genome, 67.32% in anti-genome). Blue: genome, red: anti-genome.

To comprehensively understand vsiRNAs, we analyzed the size, polar distribution and base bias of vsiRNAs (Figure 4). The vsiRNAs were mostly 19~24-nt long; the 22- and 21-nt sRNA species had the highest percentages, together making up more than 90% of the total (Figure 4A). We also found that the number of vsiRNAs reads from the anti-genome strand was slightly higher than from the genome strand. It was also observed that the vsiRNAs were evenly distributed in the positive and negative strands of the genome, indicating that the vsiRNAs were formed from the replication intermediate. In addition, a bias toward base A and U especially in the anti-genome was observed (52.1% in the genome, 67.3% in the anti-genome) (Figure 4B).

### Identity calculation and phylogenetic analyses with other viruses in family *Luteoviridae*

The full-length genome sequence and the predicted UTR and ORFs of WLYaV were compared with those of 26 other viruses in the family *Luteoviridae* (Table 1). The nucleotide identities ranged from 42.3 to 64.9% for the full-length genome, 17.8 to 50.0% for the 5′UTR, 33.0–60.3% for the I-UTR, and 11.1–48.4% for the 3′UTR, respectively. Moreover, the deduced amino acid sequence identities were 2.1–25.4% for ORF0, 4.5–48.9% for ORF1, 10.8–62.3% for ORF1-2 fusion, 31.1–72.7% for ORF3a, 29.7–86.2% for ORF3, 20.2–74.0% for ORF4, 29.9–66.0% for ORF3-5 fusion, respectively. It is worth emphasizing that the CP produced by ORF3 shared the highest identities among the viruses, and ORF0 had the lowest identities (Table 1). WLYaV had the closest relationship with ScYLV (64.9% for full length nucleotide and 86.2% for deduced amino acid sequences of ORF3). When the full-length genome and ORF3 deduced amino acid sequences of 10 representative ScYLV isolates (Elsayed et al., 2017) were compared with WLYaV (Table S4), the identities were 63.7~65.3% and 85.7~86.2%, respectively, below the species demarcation thresholds (90%) in the family *Luteoviridae* (Domier, 2012; Simmonds et al., 2017).

**Table 1:** Percent nucleotide and amino acid sequence identities of WLYaV and other members in *Luteoviridae*.

**Genus**	**Viruses**	Full length nt^*^	5′UTR nt^*^	I-UTR nt^*^	3′UTR nt^*^	ORF0 aa^*^	ORF1 aa^*^	ORF1-2 fusion aa^*^	**ORF3a**	ORF3 aa^*^	ORF4 aa^*^	ORF3-5 fusion aa^*^
*Polerovirus*	Beet chlorosis virus	50.5	32.8	55.6	39.1	2.1	27.0	42.4	33.3	43.1	27.4	30.5
	Beet mild yellowing virus	50.1	43.1	51.1	30.5	8.7	29.3	43.3	31.1	43.6	28.8	32.1
	Beet western yellows virus	50.4	32.8	57.9	36.1	16.3	30.1	44.7	51.1	46.1	31.3	32.0
	Carrot red leaf virus	50.3	36.2	46.8	35.6	18.7	32.1	44.8	46.7	36.4	23.1	32.2
	Cereal yellow dwarf virus-RPS	50.6	39.0	48.0	45.9	16.4	24.8	40.0	40.0	43.1	29.4	28.7
	Cereal yellow dwarf virus-RPV	49.4	25.3	45.9	42.5	16.8	28.6	42.7	48.9	44.0	30.5	31.0
	Chickpea chlorotic stunt virus	51.0	45.5	48.3	38.2	18.6	28.4	42.3	51.1	45.0	28.9	35.7
	Cotton leafroll dwarf virus	51.7	48.1	49.8	46.7	22.0	29.1	42.3	55.6	51.0	28.0	34.2
	Cucurbit aphid-borne yellows virus	50.2	25.4	55.2	37.1	10.7	31.5	44.8	55.6	44.7	27.6	33.4
	Maize yellow dwarf virus-RMV	52.9	42.3	44.7	48.4	15.6	30.7	45.6	44.4	42.3	29.4	42.3
	Melon aphid-borne yellows virus	51.0	25.4	51.7	32.9	16.0	30.0	43.7	45.8	44.1	30.4	36.0
	Pepper vein yellows virus	51.0	49.3	60.3	32.1	17.3	27.4	42.9	55.6	47.4	27.3	32.5
	Potato leafroll virus	49.0	24.7	60.3	23.6	15.9	28.5	45.0	48.9	41.4	27.5	28.9
	Suakwa aphid-borne yellows virus	49.2	31.9	47.0	35.7	2.2	25.3	37.5	43.8	43.5	25.6	34.0
	Sugarcane yellow leaf virus	64.9	50.0	60.3	47.7	25.4	48.9	62.3	72.7	86.2	74.0	66.0
	Tobacco vein distorting virus	51.2	48.5	57.1	23.6	5.3	29.0	43.6	51.1	46.9	29.6	32.9
	Turnip yellows virus	51.7	39.0	45.6	37.2	17.3	32.6	45.6	48.9	44.4	29.4	32.6
*Enamovirus*	Pea enation mosaic virus-1	45.5	17.8	38.5	39.9	4.3	19.4	29.9	NA^*^	29.7	NA^*^	29.9
*Luteovirus*	Barley yellow dwarf virus Ker-II	43.2	25.0	36.4	21.9	NA^*^	10.8	14.2	42.2	48.0	33.1	33.6
	Barley yellow dwarf virus - MAV	43.3	29.6	33.0	34.6	NA^*^	9.1	11.8	40.4	48.5	33.1	34.8
	Barley yellow dwarf virus-PAS	44.1	23.0	34.1	22.2	NA^*^	7.9	12.2	40.4	48.3	32.1	35.4
	Barley yellow dwarf virus-PAV	43.4	30.6	37.3	44.5	NA^*^	8.6	11.7	38.3	47.8	33.5	34.8
	Bean leafroll virus	43.2	22.5	35.6	18.0	NA^*^	4.5	13.2	31.9	40.6	20.2	29.6
	Rose spring dwarf-associated virus	43.8	20.1	36.5	11.1	NA^*^	8.4	11.9	43.4	42.1	26.7	32.9
	Soybean dwarf virus	42.3	26.1	43.3	17.7	NA^*^	6.4	10.8	44.4	39.1	26.2	30.1
Unassigned	Wheat yellow dwarf virus-GPV	51.0	39.6	46.3	27.6	11.1	27.4	41.7	53.3	45.1	29.3	30.0

* *nt, Nucleotide; aa, amino acid; NA, not applicable. Virus accession numbers are listed in Table S2*.

**Figure 5:**
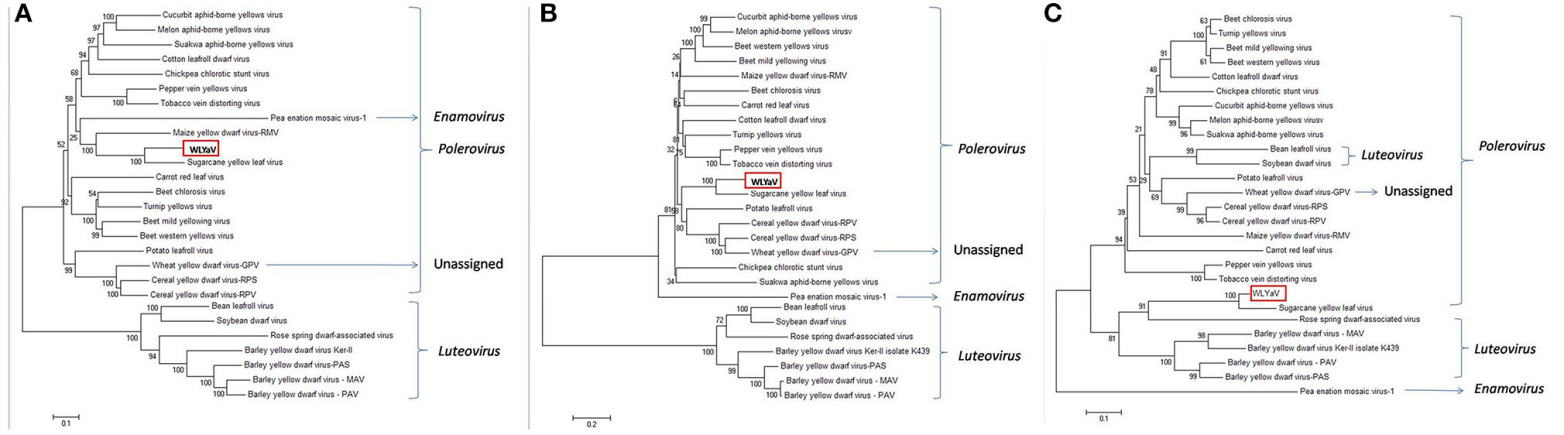
Phylogenetic analyses of WLYaV with other viruses in the family *Luteoviridae*. The phylogenetic trees were generated using the neighbor-joining method by MEGA 6 software. The percentage of replicate trees in which the associated taxa clustered together in the bootstrap test (1,000 replicates) is shown next to the branches. Phylogenetic tree based on **(A)** complete genome nucleotide sequences, deduced **(B)** RdRp and **(C)** CP amino acid sequences. The three phylogenetic trees had similar topologies, and both phylogenetic trees showed that WLYaV was most closely related to ScYLV and belonged to genus *Polerovirus*. Virus accession numbers are listed in Table S2.

The neighbor-joining phylogenetic analyses based on the nucleotide sequences of the full-length genome and deduced amino acid sequences of RdRp were performed with a bootstrap of 1,000 replications (Figure 5), which are widely used as important criteria for assigning virus species to a genus in the family *Luteoviridae*. Besides, the phylogenetic relationship of CP sequences was analyzed due to a possible recombinant site had been identified between RdRp and CP of ScYLV (Moonan et al., 2000; Smith et al., 2000). The three phylogenetic trees had similar topologies and showed that WLYaV was most closely related to ScYLV, genus *Polerovirus*. It's worth noted that in the CP tree, WLYaV, and ScYLV clustered with the members of genus *Luteovirus*, in contrast, two known luteoviruses, soybean dwarf virus (SbDV) and bean leafroll virus (BLRV) clustered with poleroviruses (Figure 5C). Therefore, a similar recombination event has probably occurred in the ancestral WLYaV genome. It could be speculated that this event may have occurred in the ancestral WLYaV and ScYLV, as similar to a drawing scenario by Domier et al. (2002) with the two luteoviruses, SbDV and BLRV. Furthermore, when the phylogenetic trees were constructed using the 10 representative isolates of ScYLV (Figure S3), WLYaV and ScYLVs were on different branches, giving the same result and indicating WLYaV and ScYLV were different. When the identities and phylogenetic analyses are considered together, this new virus could be defined as a new member of genus *Polerovirus*, family *Luteoviridae* according to criterion of the International Committee on Taxonomy of Viruses (ICTV).

### Agroinoculation infectivity assay

**Figure 6:**
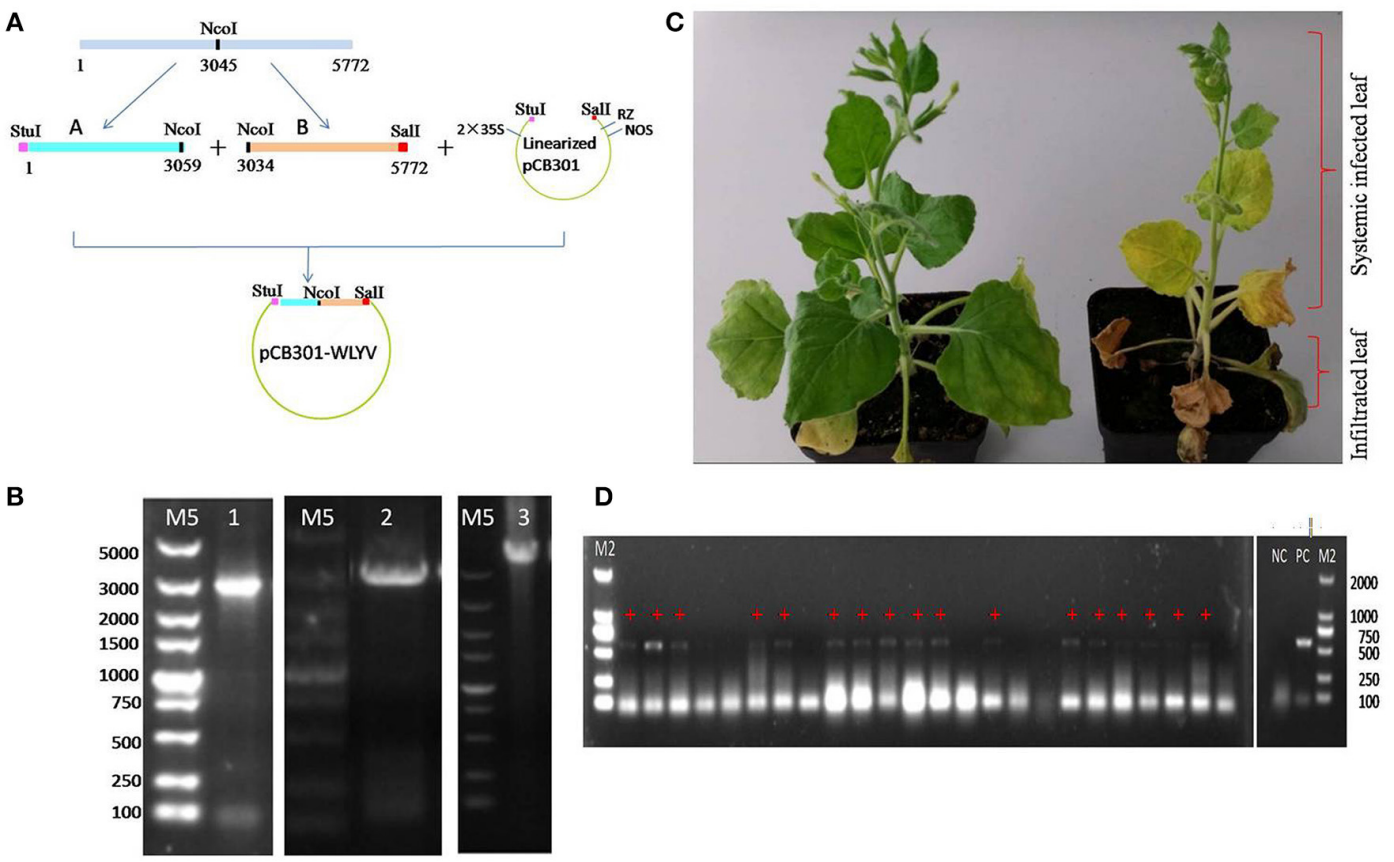
Agroinoculation infectivity assay of cDNA clone on *N. benthamiana*. **(A)** Construction of infectious cDNA clone. Whole viral genome was cloned using fragments A and B digested with *Stu*I, *Nco*I and *Sal*I and ligated with *Stu*I-*Sal*I digested pCB301 vector, 2 × 35S: promoter, RZ: ribozyme, NOS: teminator. **(B)** Confirmation of the constructs obtained by PCR, M5: DL 5 000 DNA marker, 1: pEASYT5-A, 2: pEASYT5-B, 3: pCB301-WLYaV. **(C)** Symptoms on infected *N. benthamiana* 4 weeks after infiltration of lower 3~4 leaves; upper leaves are systemically infected. Left: inoculated with pCB301 as negative control, no obvious symptoms; right: inoculated with pCB301-WLYaV, stunted plants have leaf yellowing. **(D)** Detection of WLYaV in *N. benthamiana* by RT-PCR 14 days post-inoculation, M2: DL 2 000 DNA marker, 17/24 plants were positive, NC: negative control (pCB301-inoculated *N. benthamiana*), PC: positive control (JN-U3), +: exhibiting yellowing symptom.

The full-length cDNA clone of WLYaV was constructed using vector pCB301, namely pCB301-WLYaV (Figure 6A), and confirmed by colony PCR using primers A-Stu-F and B-Sal-R (Figure 6B) and Sanger sequencing. The positive clone was used to agroinoculate 2-week-old leaves of *N. benthamiana* plants. Typical systemic symptom of leaf yellowing was observed 2 weeks later and more obvious 4 weeks later (Figure 6C). Leaves were confirmed as systemically infected using RT-PCR and subsequent sequencing of the amplification product (Figure 6D). This test was repeated three times; each time about 60–70% plants were systemically infected.

## Discussion

The recent advent of NGS, combined with bioinformatics, has accelerated the discovery and identification of virus sequences in organisms including plant, fungi and insects because it does not depend on previous information and can detect both known and unknown viruses (Kreuze et al., 2009; Wu et al., 2010; Li et al., 2012; Massart et al., 2014; Chen et al., 2015; Wang et al., 2016). More than 100 novel DNA and RNA plant viruses from different genera and families have been identified in recent years (Roossinck et al., 2015; Wu et al., 2015; Xin et al., 2017a,b). This technology also has been used to identify viruses in family *Luteoviridae*, resulting in the discovery of novel viruses such as pepper yellow leaf curl virus, citrus vein enation virus, and maize yellow mosaic virus (MaYMV) (Dombrovsky et al., 2013; Vives et al., 2013; Chen et al., 2016). Importantly, the discovery of a new viral species must be followed by its biological characterization to evaluate its impact at biosecurity, commercial, regulatory, and scientific levels (Massart et al., 2017). Our work is an example for identification of a novel virus from winter wheat sample showing dull yellowing and little dwarfing symptoms by means of sRNA sequencing technology, combined with RT-PCR and RACE-PCR to obtain the full-length genome sequence, which is the first step of its biological characterization. This virus, WLYaV, has a positive-polarity ssRNA genome with seven ORFs encoding at least seven proteins. BLAST analysis showed that WLYaV has similarities to members of the family *Luteoviridae*, and it has the highest identity with ScYLV.

Although the multiple sequence alignments of the coding and non-coding regions of the genome indicated that WLYaV had lower identities with other members in family *Luteoviridae* (described above), it has many features similar to those of members of the genus *Polerovirus*, either in genome organization or conserved sequences. Similar to other poleroviruses, WLYaV has an extra ORF0 at the 5′ terminal that is absent in luteoviruses and lacks the 3′ proximal ORF6 present in luteoviruses. The *Luteoviridae* block (ORFs 3, 4, and 5) was also found in its genome. The WLYaV and ScYLV F-box like motifs appear similar (IPIIL and VPILL, respectively) and both slightly differ from the conserved motif LPxxL/I found in other poleroviruses. The putative RdRp of WLYaV is encoded by ORF2 and it might be expressed by −1 ribosomal frameshift driven by the −1 frameshift signals at the overlap of ORF1 and 2 (nt 1,658~2,099). These include a slippery site (GGGAAAC in WLYaV, nt 1,658~1,664), which is a common feature of the viruses in family *Luteoviridae* (Krueger et al., 2013). ScYLV has the polerovirus conserved sequence ACAAAA at both of the 5′ extremity and the ends of ORF2, while WLYaV does not have this conserved sequence. Instead, there is a sequence ACUAAA respectively at the beginning of 5′ terminal and the end of ORF2 (nt 3,350–3,355) of WLYaV. Similarly, CYDV-RPS and PLRV have a conserved sequence ACAAAA at the end of ORF2, but having CAAAAC and CUUUAU respectively at the beginning of the 5′ terminal. According to the identities and phylogenetic relationships, WLYaV has the closest relationship with ScYLV, and an inter-species recombination may have occurred independently during the course of evolution and might contribute to genetic diversity of the members of family *Luteoviridae*. All the features suggested that WLYaV belongs to genus *Polerovirus*.

ScYLV, a member of the genus *Polerovirus*, family *Luteoviridae*, causes severe leaf symptoms in sugarcane and exhibits significant genetic diversity with two phylogroups (Elsayed et al., 2017). Sequences comparison and phylogenetic analyses of WLYaV with 10 representative isolates of ScYLV (Table S4, Figure S3) indicated that WLYaV and ScYLV are distinct. Considering all the results, we propose WLYaV as a new species according to the criteria in the 9th Report of the ICTV for species demarcation in the family *Luteoviridae* (Domier, 2012).

Analysis of WLYaV vsiRNAs revealed similar trend with MaYMV vsiRNAs (Chen et al., 2016), i.e., hotspots within 3′ half of the genome probably due to the production of sgRNA which is required for CP and RTD expression. The hotspots within 5′ terminal suggested the structural elements, such as stem-loops, at the 5′ terminus, and coding regions may be preferentially targeted by the host Dicer (Jaag et al., 2003; Miller et al., 2015). In *Arabidopsis thaliana*, 21-, 22- and 24-nt long siRNAs were produced by DCL4, DCL2 and DCL3, respectively (Mlotshwa et al., 2008). Most WLYaV siRNAs were 22 and 21 nt sRNAs while the total sRNAs peaked at 21 and 24 nt. Therefore, it is assumed that DCL2 and DCL4 likely play important role in generating WLYaV siRNAs whereas in most viruses and viroids, the 21 nt species is the predominant class (Donaire et al., 2009; Ma et al., 2011; Li et al., 2012; Zhang et al., 2014). For poleroviruses such as cotton leafroll dwarf virus, MaYMV, and brassica yellows virus, the abundant vsiRNA class is 22 nt (Silva et al., 2011; Chen et al., 2016; Zhou et al., 2017). This suggests that these poleroviruses and WLYaV may affect DCL4 activity which is then compensated by DCL2.

To complete the Koch's postulates for new pathogen identification, we constructed infectious cDNA clone of WLYaV, which was then used to inoculate *N. benthamiana*, similar to the method used to identify other viruses. A cDNA clone of MaYMV (genus *Polerovirus*, family *Luteoviridae*) caused systemic, symptomless infection of *N. benthamiana*, but not in maize and oat plants (Chen et al., 2016). Citrus tristeza virus (a closterovirus), which failed to agroinfect citrus plants and lacked an experimental herbaceous host, was also successfully used to agroinoculate *N. benthamiana* (Ambrós et al., 2011). In our study, at 14 d after inoculation WLYaV was detected by RT-PCR in non-inoculated leaves of the plant with symptom of yellowing. This result suggested that WLYaV can be transmitted to another host and also cause disease. For the future, the important characterization steps to evaluate the risks for the wheat production will be identifying WLYaV vector species and evaluating virus prevalence and symptomatology in wheat fields on a large scale (Massart et al., 2017). More specifically, we will collect potential vector insect species in the wheat field where the samples were collected for transmission assays in the laboratory. We will give closer attention to *Melanaphis sacchari* (sugarcane aphid) and *Rhopalosiphum maidis* (corn leaf aphid), which reportedly transmit the closest relative of WLYaV, ScYLV (Chinnaraja and Viswanathan, 2015).

In conclusion, we identified a new wheat virus that is associated with wheat leaf yellowing disease, sequenced, and analyzed its whole genome sequence and organization. Additionally, we constructed an infectious cDNA clone of WLYaV that can systemically infect plants of *N. benthamiana* and cause leaf yellowing symptoms, thus adding a step to fulfill Koch's postulates. There are still many questions about WLYaV to be answered: what are its natural vector insects, host ranges, distribution and damage in China? However, we anticipate that findings from this study will lead to a better understanding of the incidence and distribution of different wheat viruses in China, and then determine the best control measures.

## Author contributions

XW, Conceived and designed the experiments. PZ, YL, WL, and MC, Performed the experiments. PZ, WL, MC, and SM, Analyzed the data. XW, PZ, and SM, Wrote the manuscript. All authors read and approved the final manuscript.

### Conflict of interest statement

The authors declare that the research was conducted in the absence of any commercial or financial relationships that could be construed as a potential conflict of interest.

## References

[B1] Ambrós S. El-Mohtar C. Ruiz-Ruiz S. Peña L. Guerri J. Dawson W. O.. (2011). Agroinoculation of citrus tristeza virus causes systemic infection and symptoms in the presumed nonhost *Nicotiana benthamiana*. Mol. Plant Microbe Interact. 24:1119. 10.1094/MPMI-05-11-0110 21899435

[B2] Baulcombe D. (2004). RNA silencing in plants. Nature 431, 356–363. 10.1038/nature02874 15372043

[B3] Brault V. van den Heuvel J. F. Verbeek M. Ziegler-graff V. Reutenauer A. Herrbach E.. (1995). Aphid transmission of beet western yellows luteovirus requires the minor capsid read-through protein P74. EMBO J. 14, 650–659. 7882968 10.1002/j.1460-2075.1995.tb07043.x PMC398128

[B4] Chen S. Huang Q. Wu L. Qian Y. (2015). Identification and characterization of a maize-associated mastrevirus in China by deep sequencing small RNA populations. Virol. J. 12:156. 10.1186/s12985-015-0384-3 26437663 PMC4594918

[B5] Chen S. Jiang G. Wu J. Liu Y. Qian Y. Zhou X. (2016). Characterization of a novel polerovirus infecting maize in China. Viruses 8:120. 10.3390/v8050120 27136578 PMC4885075

[B6] Chinnaraja C. Viswanathan R. (2015). Quantification of sugarcane yellow leaf virus in sugarcane following transmission through aphid vector, *Melanaphis sacchari*. Virus Dis. 26, 237–242. 10.1007/s13337-015-0267-7 26645033 PMC4663716

[B7] Dombrovsky A. Glanz E. Lachman O. Sela N. Doron-Faigenboim A. Antignus Y. (2013). The complete genomic sequence of pepper yellow leaf curl virus (PYLCV) and its implications for our understanding of evolution dynamics in the genus polerovirus. PLoS ONE 8:e70722. 10.1371/journal.pone.0070722 23936244 PMC3728342

[B8] Domier L. L. (2012). Family luteoviridae, in Virus Taxonomy: Ninth Report of the International Committee on Taxonomy of Viruses, eds Andrew M. Q. K. Elliot L. Michael J. A. Carstens E. B. (San Diego, CA: Elsevier Academic Press), 1045–1053.

[B9] Domier L. L. McCoppin N. K. Larsen R. C. D'Arcy C. J. (2002). Nucleotide sequence shows that bean leafroll virus has a luteovirus-like genome organization. J. Gen. Virol. 83, 1791–1798. 10.1099/0022-1317-83-7-1791 12075101

[B10] Donaire L. Wang Y. Gonzalezibeas D. Mayer K. F. Aranda M. A. Llave C. (2009). Deep-sequencing of plant viral small RNAs reveals effective and widespread targeting of viral genomes. Virology 392, 203–214. 10.1016/j.virol.2009.07.005 19665162

[B11] Elsayed A. I. Boulila M. Odero D. C. Komor E. (2017). Phylogenetic and recombination analysis of sorghum isolates of *sugarcane yellow leaf virus*. Plant Pathol. [Epub ahead of print]. 10.1111/ppa.12708

[B12] Guilley H. Wipf-Scheibel C. Richards K. Lecoq H. Jonard G. (1994). Nucleotide sequence of cucurbit aphid-borne yellows luteovirus. Virology 202, 1012–1017. 10.1006/viro.1994.1429 8030201

[B13] Hamilton A. J. Baulcombe D. C. (1999). A species of small antisense RNA in posttranscriptional gene silencing in plants. Science 286, 950–952. 10.1126/science.286.5441.950 10542148

[B14] Hawkes J. R. Jones R. A. C. (2005). Incidence and distribution of Barley yellow dwarf virus and Cereal yellow dwarf virus in over-summering grasses in a mediterranean-type environment. Aust. J. Agric. Res. 56, 257–270. 10.1071/AR04259

[B15] Hwang Y. T. Kalischuk M. Fusaro A. F. Waterhouse P. M. Kawchuk L. (2013). Small RNA sequencing of Potato leafroll virus-infected plants reveals an additional subgenomic RNA encoding a sequence-specific RNA-binding protein. Virology 438, 61–69. 10.1016/j.virol.2012.12.012 23433865

[B16] Ian K. (2004). Gene finding in novel genomes. BMC Bioinformatics 5:59 10.1186/1471-2105-5-59 15144565 PMC421630

[B17] Jaag H. M. Kawchuk L. Rohde W. Fischer R. Emans N. Prüfer D. (2003). An unusual internal ribosomal entry site of inverted symmetry directs expression of a potato leafroll polerovirus replication-associated protein. Proc. Natl. Acad. Sci. U.S.A. 100, 8939–8944. 10.1073/pnas.1332697100 12835413 PMC166417

[B18] Jin Z. Wang X. Chang S. Zhou G. (2004). The complete nucleotide sequence and its organization of the genome of Barley yellow dwarf virus-GAV. SCI. China Ser. C Life Sci. 47, 175–182. 10.1360/03yc0076 15379250

[B19] Kozlowska-Makulska A. Guilley H. Szyndel M. S. Beuve M. Lemaire O. Herrbach E.. (2010). P0 proteins of European beet-infecting poleroviruses display variable RNA silencing suppression activity. J. Gen. Virol. 91, 1082–1091. 10.1099/vir.0.016360-0 19955562

[B20] Kreuze J. F. Perez A. Untiveros M. Quispe D. Fuentes S. Barker I.. (2009). Complete viral genome sequence and discovery of novel viruses by deep sequencing of small RNAs: a generic method for diagnosis, discovery and sequencing of viruses. Virology 388, 1–7. 10.1016/j.virol.2009.03.024 19394993

[B21] Krueger E. Beckett R. Gray S. Miller W. A. (2013). The complete nucleotide sequence of the genome of Barley yellow dwarf virus-RMV reveals it to be a new Polerovirus distantly related to other yellow dwarf viruses. Front. Microbiol. 4:205. 10.3389/fmicb.2013.00205 23888156 PMC3719023

[B22] Langmead B. Salzberg S. L. (2012). Fast gapped-read alignment with Bowtie 2. Nat. Methods 9, 357–359. 10.1038/nmeth.1923 22388286 PMC3322381

[B23] Li R. Gao S. Hernandez A. G. Wechter W. P. Fei Z. Li K. (2012). Deep sequencing of small RNAs in tomato for virus and viroid identification and strain differentiation. PLoS ONE 7:e37127. 10.1371/journal.pone.0037127 22623984 PMC3356388

[B24] Lister R. Ranieri R. (1995). Distribution and economic importance of Barley yellow dwarf, in Barley Yellow Dwarf: 40 Years of Progress, eds D'Arcy C. J. Burnett P. A. (St. Paul, MN: APS Press), 29–53.

[B25] Liu F. Wang X. Liu Y. Xie J. Gray S. M. Zhou G.. (2007). A Chinese isolate of barley yellow dwarf virus-PAV represents a third distinct species within the PAV serotype. Arch. Virol. 152, 1365–1373. 10.1007/s00705-007-0947-8 17347769

[B26] Liu X. Wu J. Wang J. Liu X. Zhao S. Li Z.. (2009). WebLab: a data-centric, knowledge-sharing bioinformatic platform. Nucleic Acids Res. 37, W33–W39. 10.1093/nar/gkp428 19465388 PMC2703900

[B27] Liu Y. Sun B. X. Zheng C. Zhou G. (2007). Three digoxigenin-labeled cDNA probes for specific detection of the natural population of Barley yellow dwarf viruses in China by dot-blot hybridization. J. Virol. Methods 145, 22–29. 10.1016/j.jviromet.2007.05.006 17561274

[B28] Luciozavaleta E. Smith D. M. Gray S. M. (2007). Variation in transmission efficiency among Barley yellow dwarf virus-RMV isolates and clones of the normally inefficient aphid vector, *Rhopalosiphum padi*. Phytopathology 91, 792–796. 10.1094/PHYTO.2001.91.8.792 18944037

[B29] Ma M. Huang Y. Gong Z. Zhuang L. Li C. Yang H.. (2011). Discovery of DNA viruses in wild-caught mosquitoes using small RNA high throughput sequencing. PLoS ONE 6:e24758. 10.1371/journal.pone.0024758 21949749 PMC3176773

[B30] Massart S. Candresse T. Gil J. Lacomme C. Predjana L. Ravnikar M.. (2017). Framework for the evaluation of biosecurity, commercial, regulatory and scientific impacts of plant viruses and viroids identified by NGS technologies. Front. Microbiol. 8:45. 10.3389/fmicb.2017.00045 28174561 PMC5258733

[B31] Massart S. Olmos A. Jijakli H. Candresse T. (2014). Current impact and future directions of high throughput sequencing in plant virus diagnostics. Virus Res. 188, 90–96. 10.1016/j.virusres.2014.03.029 24717426

[B32] Mehta Y. R. (ed.). (2014). Wheat and wheat production constraints, in Wheat Diseases and their Management (Cham: Springer Press), 1–16.

[B33] Mierlo J. T. V. Cleef K. W. R. V. Rij R. P. V. (2010). Small silencing RNAs: piecing together a viral genome. Cell Host Microbe 7, 87–89. 10.1016/j.chom.2010.02.001 20159613

[B34] Miller W. A. Rasochová L. (1997). Barley yellow dwarf viruses. Annu. Rev. Phytopathol. 35, 167–190. 10.1146/annurev.phyto.35.1.167 15012520

[B35] Miller W. A. Dinesh-Kumar S. P. Paul C. P. (1995). Luteovirus gene expression. Crit. Rev. Plant Sci. 14, 179–211. 10.1080/07352689509701926

[B36] Miller W. A. Jackson J. Feng Y. (2015). Cis- and trans-regulation of luteovirus gene expression by the 3' end of the viral genome. Virus Res. 206, 37–45. 10.1016/j.virusres.2015.03.009 25858272 PMC4722956

[B37] Miller W. A. Liu S. Beckett R. (2002). Barley yellow dwarf virus: Luteoviridae or Tombusviridae? Mol. Plant Pathol. 3, 177–183. 10.1046/j.1364-3703.2002.00112.x 20569325

[B38] Mlotshwa S. Pruss G. J. Vance V. (2008). Small RNAs in viral infection and host defense. Trends Plant Sci. 13, 375–382. 10.1016/j.tplants.2008.04.009 18550416

[B39] Mo X. Chen Z. Chen J. (2010). Complete nucleotide sequence and genome organization of a Chinese isolate of Tobacco vein distorting virus. Virus Genes 41, 425–431. 10.1007/s11262-010-0524-1 20740310

[B40] Moonan F. Molina J. Mirkov T. E. (2000). Sugarcane yellow leaf virus: an emerging virus that has evolved by recombination between luteoviral and poleroviral ancestors. Virology 269, 156–171. 10.1006/viro.1999.0162 10725208

[B41] Nickel H. Kawchuk L. Twyman R. M. Zimmermann S. Junghans H. Winter S.. (2008). Plantibody-mediated inhibition of the Potato leafroll virus P1 protein reduces virus accumulation. Virus Res. 136, 140–145. 10.1016/j.virusres.2008.05.001 18573562

[B42] Nixon P. L. Rangan A. Kim Y. G. Rich A. Hoffman D. W. Hennig M.. (2002). Solution structure of a Luteoviral P1-P2 frameshifting mRNA pseudoknot. J. Mol. Biol. 322, 621–633. 10.1016/S0022-2836(02)00779-9 12225754

[B43] Pazhouhandeh M. Dieterle M. Marrocco K. Lechner E. Berry B. Brault V.. (2006). F-box-like domain in the polerovirus protein P0 is required for silencing suppressor function. Proc. Natl. Acad. Sci. U.S.A. 103, 1994–1999. 10.1073/pnas.0510784103 16446454 PMC1413668

[B44] Pfeffer S. Dunoyer P. Heim F. Richards K. E. Jonard G. Ziegler-Graff V. (2002). P0 of beet western yellows virus is a suppressor of posttranscriptional gene silencing. J. Virol. 76, 6815–6824. 10.1128/JVI.76.13.6815-6824.2002 12050394 PMC136274

[B45] Pfeffer S. Dunoyer P. Heim F. Richards K. E. Jonard G. Zieglergraff V. (2017). Retraction for pfeffer et al. P0 of beet western yellows virus is a suppressor of posttranscriptional gene silencing. J. Virol. 91:e00022–17. 10.1128/JVI.00022-17

[B46] Prüfer D. Tacke E. Schmitz J. Kull B. Kaufmann A. Rohde W. (1992). Ribosomal frameshifting in plants: a novel signal directs the -1 frameshift in the synthesis of the putative viral replicase of potato leafroll luteovirus. EMBO J. 11, 1111–1117. 1547775 10.1002/j.1460-2075.1992.tb05151.x PMC556553

[B47] Rochow W. F. (1969). Biological properties of four isolates of barley yellow dwarf virus. Phytopathology 59, 1580–1589. 5377734

[B48] Rochow W. F. Muller I. (1971). A fifth variant of barley yellow dwarf virus in New York. Plant Dis. 55, 874–877.

[B49] Roossinck M. J. Martin D. P. Roumagnac P. (2015). Plant virus metagenomics: advances in virus discovery. Phytopathology 105, 716–727. 10.1094/PHYTO-12-14-0356-RVW 26056847

[B50] Rotenberg D. Bockus W. W. Whitfield A. E. Hervey K. Baker K. D. Ou Z.. (2016). Occurrence of viruses and associated grain yields of paired symptomatic and nonsymptomatic tillers in Kansas winter wheat fields. Phytopathology 106, 202–210. 10.1094/PHYTO-04-15-0089-R 26799958

[B51] Shen Y. Zhao X. Yao M. Li C. Miriam K. Xue Z.. (2014). A versatile complementation assay for cell-to-cell and long distance movements by cucumber mosaic virus based agro-infiltration. Virus Res. 190, 25–33. 10.1016/j.virusres.2014.06.013 25014544

[B52] Silva T. F. Romanel E. A. Andrade R. R. Farinelli L. Østerås M. Deluen C.. (2011). Profile of small interfering RNAs from cotton plants infected with the polerovirus cotton leafroll dwarf virus. BMC Mol. Biol. 12:40. 10.1186/1471-2199-12-40 21864377 PMC3189115

[B53] Simmonds P. Adams M. J. Benko M. Breitbart M. Brister J. R. Carstens E. B.. (2017). Consensus statement: virus taxonomy in the age of metagenomics. Nat. Rev. Microbiol. 15, 161–168. 10.1038/nrmicro.2016.177 28134265

[B54] Smirnova E. Firth A. E. Miller W. A. Scheidecker D. Brault V. Reinbold C.. (2015). Discovery of a small non-AUG-initiated ORF in poleroviruses and luteoviruses that is required for long-distance movement. PLoS Pathog. 11:e1004868. 10.1371/journal.ppat.1004868 25946037 PMC4422679

[B55] Smith G. R. Borg Z. Lockhart B. E. Braithwaite K. S. Gibbs M. J. (2000). Sugarcane yellow leaf virus: a novel member of the Luteoviridae that probably arose by inter-species recombination. J. Gen. Virol. 81, 1865–1869. 10.1099/0022-1317-81-7-1865 10859394

[B56] Tamura K. Stecher G. Peterson D. Filipski A. Kumar S. (2013). MEGA6: molecular evolutionary genetics analysis version 6.0. Mol. Biol. Evol. 30, 2725–2729. 10.1093/molbev/mst197 24132122 PMC3840312

[B57] Vives M. C. Velázquez K. Pina J. A. Moreno P. Guerri J. Navarro L. (2013). Identification of a new enamovirus associated with citrus vein enation disease by deep sequencing of small RNAs. Phytopathology 103, 1077–1086. 10.1094/PHYTO-03-13-0068-R 23718835

[B58] Voinnet O. (2001). RNA silencing as a plant immune system against viruses. Trends Genet. 17, 449–459. 10.1016/S0168-9525(01)02367-8 11485817

[B59] Wang Q. Ma X. Qian S. Zhou X. Sun K. Chen X.. (2015). Rescue of a plant negative-strand RNA virus from cloned cDNA: insights into enveloped plant virus movement and morphogenesis. PLoS Pathog. 11:e1005223. 10.1371/journal.ppat.1005223 26484673 PMC4616665

[B60] Wang S. Zhang C. Cheng R. Yu X. Lu J. (2016). A Cripavirus in the brown planthopper, *Nilaparvata lugens*. J. Gen. Virol. 97, 706–714. 10.1099/jgv.0.000394 26746854

[B61] Wang X. Liu Y. Han C. Wu Y. Zhao Z. (2010). Present situation and developing strategies for the research and control of wheat viral diseases. Plant Prot. 36, 13–19. 10.3969/j.issn.0529-1542.2010.03.004

[B62] Wu B. Blanchardletort A. Liu Y. Zhou G. Wang X. Elena S. F. (2011). Dynamics of molecular evolution and phylogeography of Barley yellow dwarf virus-PAV. PLoS ONE 6:e16896. 10.1371/journal.pone.0016896 21326861 PMC3033904

[B63] Wu Q. Ding S. Zhang Y. Zhu S. (2015). Identification of viruses and viroids by next-generation sequencing and homology-dependent and homology-independent algorithms. Ann. Rev. Phytopathol. 53, 425–444. 10.1146/annurev-phyto-080614-120030 26047558

[B64] Wu Q. Luo Y. Lu R. Lau N. Lai E. C. Li W. X.. (2010). Virus discovery by deep sequencing and assembly of virus-derived small silencing RNAs. Proc. Natl. Acad. Sci. U.S.A. 107, 1606–1611. 10.1073/pnas.0911353107 20080648 PMC2824396

[B65] Xin M. Cao M. Liu W. Ren Y. Lu C. Wang X. (2017a). The genomic and biological characterization of *Citrullus lanatus* cryptic virus infecting watermelon in China. Virus Res. 232, 106–112. 10.1016/j.virusres.2017.02.009 28238875

[B66] Xin M. Cao M. Liu W. Ren Y. Zhou X. Wang X. (2017b). Two negative-strand RNA viruses identified in watermelon represent a novel clade in the order Bunyavirales. Front. Microbiol. 8:1514. 10.3389/fmicb.2017.01514 28848524 PMC5552725

[B67] Zerbino D. R. Birney E. (2008). Velvet: algorithms for de novo short read assembly using de Bruijn graphs. Genome Res. 18, 821–829. 10.1101/gr.074492.107 18349386 PMC2336801

[B68] Zhang W. Cheng Z. Xu L. Wu M. Waterhouse P. Zhou G.. (2009). The complete nucleotide sequence of the barley yellow dwarf GPV isolate from China shows that it is a new member of the genus Polerovirus. Arch. Virol. 154, 1125–1128. 10.1007/s00705-009-0415-8 19551470

[B69] Zhang Z. Qi S. Tang N. Zhang X. Chen S. Zhu P.. (2014). Discovery of replicating circular RNAs by RNA-seq and computational algorithms. PLoS Pathog. 10:e1004553. 10.1371/journal.ppat.1004553 25503469 PMC4263765

[B70] Zhao K. Liu Y. Wang X. (2010). Reverse transcription loop-mediated isothermal amplification of DNA for detection of barley yellow dwarf viruses in China. J. Virol. Methods 169, 211–214. 10.1016/j.jviromet.2010.06.020 20637237

[B71] Zhou C. Zhang X. Liu S. Wang Y. Li D. Yu J. L.. (2017). Synergistic infection of BrYV and PEMV 2 increases the accumulations of both BrYV and BrYV-derived siRNAs in *Nicotiana benthamiana*. Sci. Rep. 7:45132. 10.1038/srep45132 28345652 PMC5366869

[B72] Zhou G. Zhang S. Qian Y. (1987). Identification and application of four strains of wheat yellow dwarf virus. Sci. Agri. Sin. 298, 434–440.

