# Supplementary material for: Identification, Characterization and Full-Length Sequence Analysis of a Novel Polerovirus Associated with Wheat Leaf Yellowing Disease
Source: Front Microbiol. 2017 Sep 6;8:1689. doi: 10.3389/fmicb.2017.01689 (PMC5592212; doi:10.3389/fmicb.2017.01689)
Supplement: Supplementary file 1 [file Presentation1.pdf]

Supplementary Materials

Identification, Characterization and Full-Length Sequence Analysis of a Novel Polerovirus Associated with Wheat Leaf Yellowing Disease

Peipei Zhang, Yan Liu, Wenwen Liu, Mengji Cao, Sebastien Massart*, Xifeng Wang*

*Corresponding author: xfwang@ippcaas.cn; sebastien.massart@ulg.ac.be

TABLE S1 | All primers used in this study

Function	Primer	Sequence (5′ to 3′)	Size (bp)
Detection of known viruses	GAV-F	ATGAATTCAGTAGGCCGTAGAA	960
	GAV-R	GTCTCGGTTTCCTCCAATGTG	
	GPV-F	GTAGACGCGGAACCCGGT	920
	GPV-R	AAAGACGAGTTCCGCCGAAG	
	PAV-F	ATGAATTCAGTAGGCCGTAGGGGAC	501
	PAV-R	TCACCGATGTCGCCTGGAAC	
Contig validation	1F	GGAAAGCCTGCTCTGGAC	928
	1R	GTGGTCGGCAATTTCTTC	
	2F	CACTGGTGGGAAATGGTC	509
	2R	ACTTGTCTGTTCGGCTTGT	
	3F	GGGAACCGCACAAGCAGA	571
	3R	GGAGACTCAAGCGCATCG	
	4F	GGACCTGGAGGAAGAAGC	989
	4R	CCATCACCTGTTGCGTCT	
Fill gaps	1-2F	TATTGGAACGGCAAGACC	262
	1-2R	TTTCCCACCAGTGTTTACC	
	2-3F	TCATCTCAGAGCACCCAG	658
	2-3R	TCTGTGGATAAGCCGAAT	
	3-4F	ATGGAGGTGCGTAATGCC	619
	3-4R	CAGATTTACCACGATTTGAC	
RACE	5′-GSP1	CCACAGGGTTGTCTTC	
	5′-GSP2	CGCTCAAGAGGCAAAATGTC	
	5′-GSP3	TCGCCATAGGTTAGATTCAA	
	3′-GSP2	GATTCCAAAGTGGCGGGTCA	
	3′-GSP3	AGACGCAACAGGTGATGGGT	
Infectious cDNA clone construction	A-Stu-F	aggcctGACTAAAGAAACTACGGGGGA	3059
	A-Nco-R	CATCGTCTCccatggCTATCGCCCA	
	B-Nco-F	TGGGCGATAGccatggGAGACGATG	2739
	B-Sal-R	gtcgacGTTATCTTTTTACTAGGAA	
Detection of WLYaV	CP-F	ATGAATACGGGAGGTAACCGCA	570
	CP-R	CTATTTAGGGTTCTGGAATTGA	

F: forward

R: reverse

TABLES2 | Virus abbreviations and GenBank accession numbers used for identity and phylogenetic analyses in this study

Genus	Virus	Abbreviation	GenBank Accession No.
Polerovirus	Beet chlorosis virus	BChV	NC_002766.1
	Beet mild yellowing virus	BMYV	NC_003491.1
	Beet western yellows virus	BWYV	NC_004756.1
	Carrot red leaf virus	CtRLV	NC_006265.1
	Cereal yellow dwarf virus-RPS	CYDV-RPS	NC_002198.2
	Cereal yellow dwarf virus-RPV	CYDV-RPV	NC_004751.1
	Chickpea chlorotic stunt virus	CpCSV	NC_008249.1
	Cotton leafroll dwarf virus	CoLRDV	NC_014545.1
	Cucurbit aphid-borne yellows virus	CABYV	NC_003688.1
	Maize yellow dwarf virus-RMV	MYDV-RMV	NC_021484.1
	Melon aphid-borne yellows virus	MABYV	NC_010809.1
	Pepper vein yellows virus	PVYV	NC_015050.1
	Potato leafroll virus	PLRV	NC_001747.1
	Suakwa aphid-borne yellows virus	SwABYV	NC_018571.2
	Sugarcane yellow leaf virus	ScYLV	NC_000874.1
	Tobacco vein distorting virus	TVDV	NC_010732.1
	Turnip yellows virus	TuYV	NC_003743.1
Enamovirus	Pea enation mosaic virus-1	PEMV-1	NC_003629.1
Luteovirus	Barley yellow dwarf virus Ker-II	BYDV	NC_021481.1
	Barley yellow dwarf virus - MAV	BYDV-MAV	NC_003680.1
	Barley yellow dwarf virus-PAS	BYDV-PAS	NC_002160.2
	Barley yellow dwarf virus-PAV	BYDV-PAV	NC_004750.1
	Bean leafroll virus	BLRV	NC_003369.1
	Rose spring dwarf-associated virus	RSDaV	NC_010806.1
	Soybean dwarf virus	SbDV	NC_003056.1
Unassigned	Wheat yellow dwarf virus-GPV	WYDV-GPV	NC_012931.1

TABLE S3 | Detection of known virus in the wheat samples collected from Jinan, China by RT-PCR

Sample	BYDV-GAV	BYDV-PAV	WYDV-GPV	WLYaV
1		√		
2	√			
3				√
4		√		
5				
6	√			
7			√	
8				
9		√		
10	√			
11		√		
12				

√: Positive

TABLE S4 | Identities (%) of WLYaV with ten representative ScYLV isolates

Isolate	Accession No.	Full-length	ORF3
		nt	aa
REU-YL1a	AM072754.1	63.8	86.2
IND	AY236971.1	65.0	86.2
PER-YL1a	AM072752.1	63.7	85.7
BRA-YL1	AM072750.1	63.8	85.2
Haw87-4094	GU570006.1	65.3	86.2
CBLK97154	KF680098.1	65.1	85.7
CHN1	GU327735.1	64.7	85.7
CHN-GD-ZJ15	HQ245320.1	64.6	86.2
GZ-GZ18	KF477092.1	65.1	85.2
USA	NC_000874.1	64.9	86.2

nt: nucleotide; aa: amino acid

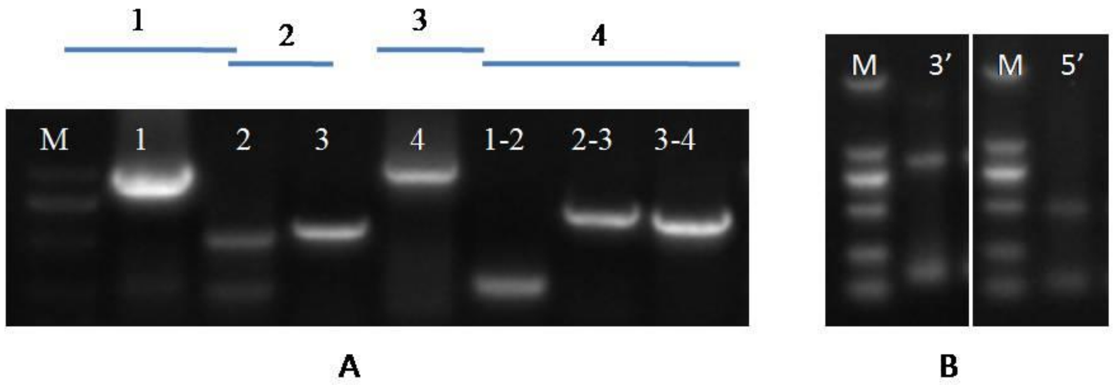

FIGURE S1 | Completion of full-length genome of wheat leaf yellowing associated virus (WLYaV) by RT-PCR and RACE-PCR. (A) Confirmation of assembled contigs and gap filling by RT-PCR, blue bars above the electrophoretogram: 4 contigs having high identities with viruses in *Luteoviridae* with the length of 1 151, 701, 718 and 1 714 nt respectively, the putative relative positions of the contigs were determined by aligning the contigs with their closest viral genome (sugarcane yellow leaf virus (ScYLV), AF157029.1), M: DL 2,000 DNA marker, 1 to 4 correspond to the 4 assembled contigs, 1-2, 2-3 and 3-4 correspond to amplicons produced to fill the gaps and had more than 70 nt overlap; **(B)** Analysis of terminal sequences by 5′ and 3′ RACE. Finally, all the PCR products were sequenced and assembled to full-length genome of WLYaV using DNAMAN program (V.6).

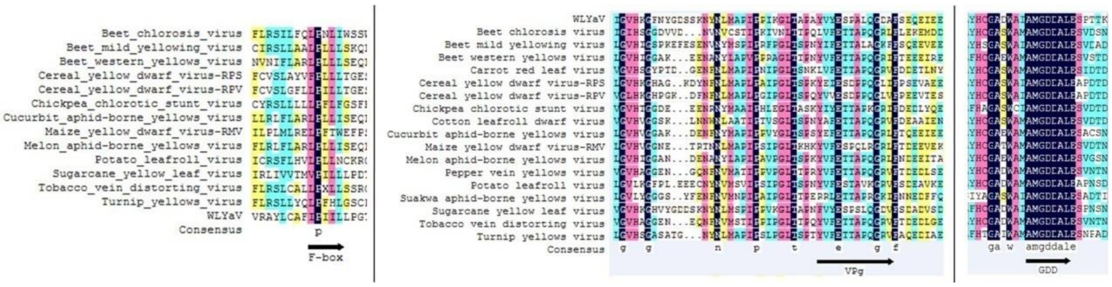

FIGURE S2 | Multiple alignments of amino acid sequences of P0, P1 and P2 from several poleroviruses. The positions of the F-box, VPg and RNA dependent RNA polymerase signature GDD are highlighted with arrows. F-box Motif in ORF0: LPxxL/I, which is conserved in the poleroviruses. VPg Motif in ORF 1: the Glu residue (e) corresponds to the protease cleavage site. GDD Motif in ORF 2: is conserved in family *Luteoviridae*.

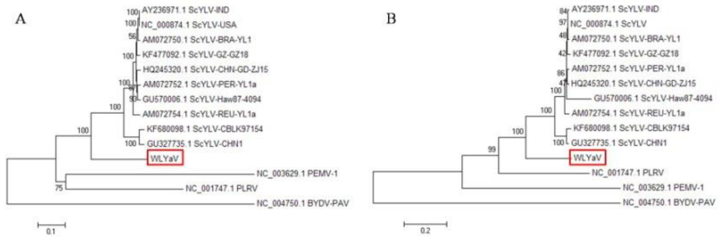

FIGURE S3 | Phylogenetic analysis of WLYaV with ten representative ScYLV isolates. Phylogenetic tree of **(A)** complete genome nucleotide sequences, deduced **(B)** RdRp and **(C)** CP amino acid sequences.
